# Multicellular immunosuppressive networks in microsatellite-stable colorectal cancer: CD8+ T cell exclusion, dysfunction, exhaustion, and therapeutic implications

**DOI:** 10.3389/fimmu.2026.1945393

**Published:** 2026-08-25

**Authors:** Zhiyuan Xia, Haining Chen, Yefu Shen, Minghui Lin, Xihua Xie, Hao Shu, Shen Pang, Dongchao Liu, Yunfei Cao, Sen Zhang

**Affiliations:** 1 Department of Colorectal & Anal Surgery, The First Affiliated Hospital of Guangxi Medical University, Nanning, China; 2 Guangxi Key Laboratory of Enhanced Recovery After Surgery for Gastrointestinal Cancer, Nanning, China

**Keywords:** CD8+ T cell exhaustion, combination immunotherapy, microsatellite-stable colorectal cancer, multicellular immunosuppressive network, tumor microenvironment spatial architecture

## Abstract

Microsatellite-stable (MSS) colorectal cancer (CRC), comprising ~85–90% of cases, remains almost universally refractory to PD-1/PD-L1 monotherapy (objective response rate 5–10%), in stark contrast to the marked efficacy of immune checkpoint inhibitors (ICIs) in microsatellite-instability-high (MSI-H) disease. Here we synthesize evidence that CD8^+^ T cell exclusion, dysfunction, and exhaustion in MSS CRC are orchestrated not by cell-autonomous failures but by a multicellular immunosuppressive network in which tumor-associated macrophages (TAMs), cancer-associated fibroblasts (CAFs), regulatory T cells (Tregs), and myeloid-derived suppressor cells (MDSCs) cooperate through mechanistically distinct yet spatially integrated axes. We propose a four-layer hierarchical model (physical exclusion, effector dysfunction, metabolic deprivation, and active suppression) that culminates in a stereotyped three-layer spatial niche best documented in CRC liver metastases (outer mCAF belt, Tex/TSTR band, inner SPP1^+^ TAM aggregates). MSS-associated mechanisms include the TAM-derived COX2/PGE2/TIGIT axis driving terminal exhaustion, a functional IL-10^-^/IL-10^+^ Treg dichotomy, and CAF-mediated suppression of dendritic-cell CXCL9/10 production that blocks CXCR3^+^ CD8 recruitment. Effective therapeutic strategies must therefore target multiple layers simultaneously, including TAM reprogramming, stromal remodeling, precision Treg depletion, and MDSC differentiation, rather than relying on additional checkpoint inhibitors, which have proven insufficient as monotherapy or in dual combinations.

## 1 Introduction

Colorectal cancer (CRC) remains one of the most prevalent and lethal malignancies worldwide. According to GLOBOCAN 2020 estimates, approximately 1.93 million new CRC cases were diagnosed globally, accounting for 10.0% of all incident cancers, while nearly 935,000 deaths were attributed to the disease - representing 9.4% of all cancer mortality and ranking CRC as the second leading cause of cancer death (1). Despite advances in surgical techniques, cytotoxic chemotherapy, and molecularly targeted agents, the prognosis of advanced and metastatic CRC remains poor. A critical determinant of clinical outcome is the mismatch repair (MMR) status of the tumor. Approximately 10–15% of CRC cases harbor deficient MMR (dMMR) or microsatellite instability-high (MSI-H) phenotypes, characterized by high tumor mutational burden (TMB) and robust immune infiltration. The remaining 85–90% are proficient MMR (pMMR) or microsatellite-stable (MSS) tumors, which display low TMB, sparse T cell infiltration, and a profoundly immunosuppressive tumor microenvironment (TME) (2). This molecular dichotomy has profound therapeutic implications that have fundamentally reshaped the landscape of CRC immunotherapy.

The advent of immune checkpoint inhibitors (ICIs) targeting the programmed death-1 (PD-1)/PD-L1 axis has transformed the treatment of MSI-H/dMMR CRC. In the landmark KEYNOTE-177 trial, pembrolizumab demonstrated superior efficacy over standard chemotherapy as first-line therapy for MSI-H/dMMR metastatic CRC, achieving a median progression-free survival of 16.5 months versus 8.2 months (hazard ratio 0.60; 95% CI 0.45–0.80), leading to regulatory approval in this setting (3). Earlier phase II data with nivolumab in previously treated MSI-H/dMMR CRC had established the proof-of-concept for PD-1 blockade in this population (4). The mechanistic basis for this sensitivity lies in the exceptionally high neoantigen burden of dMMR tumors - averaging 1,782 somatic mutations compared with 73 in pMMR counterparts (P = 0.007) - which fuels robust antigen-specific T cell responses that are reinvigorated upon checkpoint blockade (5). By contrast, MSS CRC is almost universally refractory to PD-1/PD-L1 monotherapy, with objective response rates of only 5–10% (2). This stark disparity defines one of the most pressing unmet needs in oncology: understanding and overcoming the mechanisms of immune resistance in MSS CRC.

Central to the prognostic landscape of CRC is the functional state of tumor-infiltrating CD8+ cytotoxic T lymphocytes. The Immunoscore, a standardized quantification of CD3+ and CD8+ T cell densities at the tumor core and invasive margin, was first established as a prognostic tool in a seminal study demonstrating that the type, density, and location of immune cells within CRC tumors predicted clinical outcome more accurately than conventional histopathological staging (6). Subsequent international validation across 3,539 patients from 14 centers in 13 countries confirmed the Immunoscore as the strongest independent prognostic factor in CRC, outperforming conventional TNM staging (7). Patients with high Immunoscore exhibit a five-year recurrence rate of only 8%, compared with 32% in those with low Immunoscore (hazard ratio 0.20), underscoring the paramount importance of cytotoxic T cell activity in disease control. Critically, however, the mere presence of CD8+ T cells is insufficient; their functional state is a critical determinant of anti-tumor activity. In MSS CRC, CD8+ T cells are frequently present yet functionally impaired - a state of progressive dysfunction known as T cell exhaustion - rendering them incapable of mounting effective anti-tumor responses despite their physical proximity to malignant cells.

T cell exhaustion is a distinct differentiation state arising from chronic antigen exposure or sustained immunosuppressive signals, defined by the hierarchical loss of effector functions - beginning with interleukin-2 (IL-2) production and proliferative capacity, followed by tumor necrosis factor-α (TNF-α) secretion, and culminating in the loss of interferon-γ (IFN-γ) production and cytolytic activity - alongside the co-expression of multiple inhibitory receptors including PD-1, TIM-3, LAG-3, TIGIT, and CD39 (8). Exhaustion is epigenetically stabilized and represents a fundamentally distinct transcriptional state from functional effector or memory T cells, explaining why it cannot be reversed by simply removing the antigen or blocking a single inhibitory receptor. In MSS CRC, exhaustion exhibits unique features that distinguish it from the antigen-driven exhaustion observed in MSI-H tumors or chronic viral infections. Specifically, MSS CRC is characterized by a predominance of PD-1+TIGIT+ terminally exhausted CD8+ T cells - a phenotype largely absent in MSI-H tumors, where PD-1+ T cells retain cytokine production and cytotoxic activity (9). This MSS-specific terminal exhaustion is not driven by high neoantigen burden alone but rather is strongly shaped by a complex network of cellular interactions within the TME, explaining why single-agent PD-1 blockade fails to restore T cell function.

Emerging evidence has revealed that CD8+ T cell exclusion, dysfunction, and exhaustion in MSS CRC are orchestrated by a multicellular immunosuppressive network comprising four major non-malignant cell populations: tumor-associated macrophages (TAMs), cancer-associated fibroblasts (CAFs), regulatory T cells (Tregs), and myeloid-derived suppressor cells (MDSCs) (9–11). These cell types do not act in isolation; rather, they engage in extensive bidirectional crosstalk that amplifies immunosuppression across multiple layers - from the physical exclusion of T cells from the tumor parenchyma, to the direct impairment of T cell effector function, to the metabolic deprivation of T cells within the TME. The cancer hallmarks framework has increasingly recognized non-cell-autonomous immune evasion - mediated in part by stromal and myeloid networks - as a defining feature of cancer progression and therapeutic resistance (12). Despite the clinical urgency, the mechanisms by which these multicellular networks cooperatively drive CD8+ T cell exclusion, dysfunction, and exhaustion in MSS CRC, and the spatial architecture through which this immunosuppressive ecosystem is organized, remain incompletely integrated.

In this Review, we provide a mechanistic framework for understanding how multicellular immunosuppressive networks orchestrate CD8+ T cell exclusion, dysfunction, and exhaustion in MSS CRC. We first delineate the landscape of T cell exhaustion in CRC, with emphasis on the MSS-specific features that distinguish it from MSI-H disease. We then systematically examine the molecular mechanisms by which each of the four major cellular axes - TAM, CAF, Treg, and MDSC - drives T cell exclusion, effector impairment, metabolic suppression, and terminal exhaustion, before integrating these axes into a unified model of the immunosuppressive network and its spatial organization. Finally, we discuss therapeutic strategies that target this multicellular network, with the goal of providing a rational basis for the design of combination immunotherapy regimens capable of overcoming the profound immune resistance of MSS CRC.

## 2 CD8+ T cell exclusion, dysfunction, and exhaustion in CRC: landscape and MSS-specific features

### 2.1 Molecular definition and differentiation hierarchy of T cell exhaustion

T cell exhaustion was first described as a state of progressive dysfunction arising during chronic viral infection, characterized by the hierarchical loss of effector functions and the sustained co-expression of multiple inhibitory receptors (8). Comprehensive reviews have since established exhaustion as a conserved differentiation state across chronic infections and cancer, governed by a core transcriptional and epigenetic program (13, 14). The functional deterioration follows a defined sequence: IL-2 production and high proliferative capacity are lost first, followed by TNF-α secretion, and finally IFN-γ production and cytolytic activity - a hierarchy that reflects the graded severity of exhaustion across different disease contexts (8). At the phenotypic level, exhausted CD8+ T cells are defined by the co-expression of inhibitory receptors including PD-1, TIM-3, LAG-3, TIGIT, and CD39, alongside reduced expression of effector molecules such as granzyme B (GZMB) and perforin. Crucially, exhaustion is not a reversible state of functional downregulation but rather an epigenetically stabilized differentiation program that is transcriptionally and chromatinically distinct from functional effector or memory T cells - a distinction with profound implications for therapeutic intervention.

The transcription factor TOX (thymocyte selection-associated high mobility group box protein) has emerged as the master regulator of the exhaustion program. TOX expression is induced by high-quantity, sustained TCR signaling - driven by antigen load rather than antigen affinity - and its levels correlate positively with PD-1 expression across multiple disease settings (15, 16). Mechanistically, TOX orchestrates exhaustion through a dual transcriptional and epigenetic mechanism: it opens chromatin at exhaustion-specific loci while simultaneously closing regions associated with effector function, thereby locking cells into the exhausted state (16). A companion study published simultaneously confirmed that TOX is required for the establishment of the exhaustion-specific epigenetic landscape and that its deletion prevents the acquisition of the exhausted chromatin state (17). Critically, the epigenetic imprint imposed by TOX is remarkably stable: exhausted T cells that have undergone TOX-driven chromatin remodeling fail to fully revert to a durable functional state even after antigen removal or checkpoint blockade, explaining the limited reversibility of exhaustion in chronic disease settings (18). Genetic deletion of TOX (ToxΔ) in chronic infection models initially yields T cells with enhanced effector function - elevated TNF-α and IFN-γ production - but at the cost of a dramatic reduction in the TCF1+ progenitor pool, ultimately leading to clonal collapse and failure of long-term viral control (15). This finding establishes TOX as a paradoxical “exhaustion lock” that, while limiting effector function, is simultaneously required for the maintenance of the progenitor compartment that sustains the exhausted T cell pool. For MSS CRC, this framework implies that durable reversal of exhaustion is unlikely to be achieved by blocking a single inhibitory receptor once a stable exhausted chromatin state has been established.

Within the exhausted CD8+ T cell compartment, two functionally distinct subpopulations have been identified that differ fundamentally in their therapeutic relevance. Progenitor exhausted T cells (Tpex), defined by expression of TCF1 (encoded by TCF7), SELL, and SLAMF6 with low expression of TIM-3, CD39, and CD101, retain self-renewal capacity and the ability to generate effector progeny upon PD-1 blockade. This TCF1+ progenitor population was independently identified in chronic LCMV infection and tumor models as the primary responder to checkpoint blockade (19, 20). Terminal exhausted T cells (Ttex), by contrast, are TCF1−PD-1hi and co-express TIM-3, LAG-3, and TIGIT; they display markedly reduced cytotoxic potential and limited responsiveness to checkpoint inhibition. Recent work has further refined the developmental origin of this hierarchy: the transcriptional regulator ID3 (inhibitor of DNA binding 3) marks a population of stem-like memory T cells during acute infection that serve as the exclusive precursors of Tpex cells in the context of chronic antigen exposure or cancer (21). ID3 expression itself confers the capacity to sustain T cell responses over time, establishing a complete developmental axis from ID3+ stem-like memory T cells through Tpex to Ttex that is now recognized as the fundamental organizational principle of the exhausted T cell compartment.

### 2.2 Clinical significance of exhaustion subsets in CRC

The Tpex/Ttex hierarchy carries direct prognostic and predictive significance in CRC. Multiplex immunohistochemistry (mIHC) quantification of Tpex cells (TCF1+PD-1+CD8+) in CRC tissue demonstrates a positive correlation between Tpex density and overall survival, consistent with the established role of this population as the primary responder to PD-1 blockade (22). Single-cell RNA sequencing (scRNA-seq) analyses have further revealed that in pMMR CRC, a subset of CD103−CD8+ T cells (termed CD103N) adopts a Tpex-like transcriptional state and shares TCR clonotypes with CD103+ effector T cells, suggesting a circulating replenishment mechanism whereby peripheral Tpex continuously seed the tumor (23). TLS may provide intra-tumoral niches that support T cell priming and maintenance of progenitor-like T cell states, and TLS density has been independently validated as a prognostic biomarker in CRC (24). Large-scale single-cell RNA sequencing of CRC tumor ecosystems has provided a high-resolution transcriptional map of CD8+ T cell states - including Tpex, intermediate, and terminal exhaustion - and their spatial relationships with immunosuppressive stromal and myeloid cells, establishing the cellular context in which exhaustion progression occurs (10). A spatiotemporal single-cell analysis of CRC tumors from patients with divergent immunotherapy responses has further decoded the cellular dynamics underlying response versus resistance, revealing that the balance between Tpex maintenance and Tpex-to-Ttex conversion appears to be associated with ICI outcome (25).

The prognostic significance of Ttex cells is more complex and context-dependent. Immunogenomic analyses of CRC cohorts have identified Ttex enrichment as a feature associated with poor prognosis in most settings, yet Ttex abundance has also been discussed as a potential predictor of improved survival in patients receiving oxaliplatin-based chemotherapy, possibly because oxaliplatin-induced immunogenic cell death (ICD) can partially reactivate anti-tumor immune programs (26). These discordant findings highlight a critical methodological challenge: the accurate identification of Ttex requires co-expression of multiple exhaustion markers, as reliance on TCF1−PD-1+ alone fails to exclude activated effector T cells and may underestimate or misclassify the true extent of terminal exhaustion, particularly in spatially heterogeneous tumors where tissue microarray (TMA) designs may inadequately sample the full spectrum of the TME.

### 2.3 MSS-specific features of CD8+ T cell dysfunction and exhaustion

**Figure 1:**
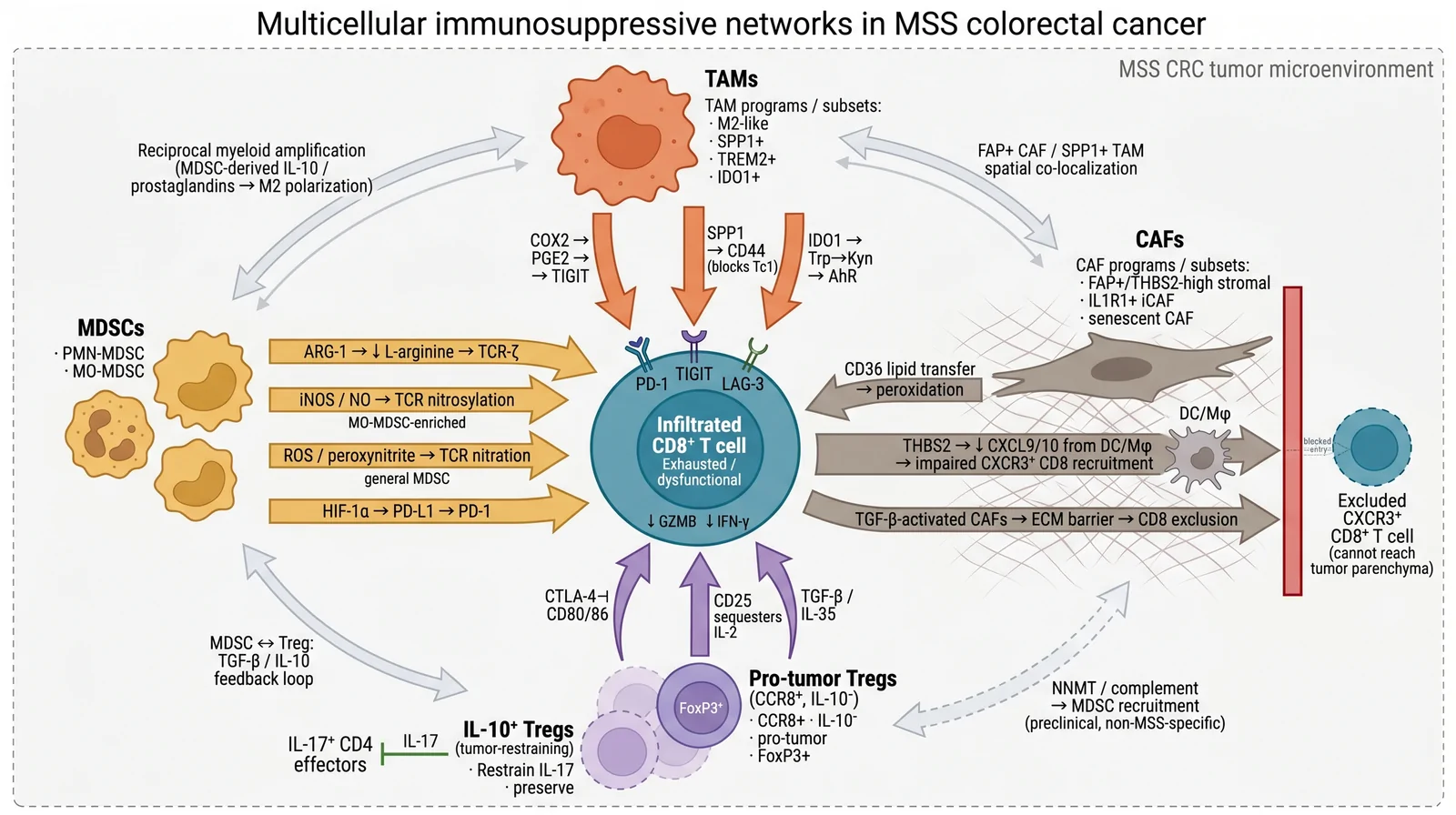
Multicellular immunosuppressive network driving CD8^+^ T cell exclusion, dysfunction, and exhaustion in microsatellite-stable colorectal cancer.

The exhaustion landscape in MSS CRC is fundamentally distinct from that observed in MSI-H disease, and this distinction has direct mechanistic and therapeutic implications. In MSI-H CRC, the high neoantigen burden drives robust antigen-specific T cell priming, generating a substantial Tpex pool that can be reinvigorated by PD-1 blockade; the resulting T cell responses are antigen-driven and, at least partially, reversible upon checkpoint inhibition. In MSS CRC, by contrast, the low TMB means that antigen-specific priming is often insufficient to sustain a comparably robust Tpex pool. Instead, the dominant exhaustion phenotype is PD-1+TIGIT+ terminal exhaustion - a phenotype that is largely absent in MSI-H tumors, where PD-1+ CD8+ T cells retain cytokine production and cytotoxic activity (9). This MSS-specific co-expression of TIGIT on exhausted T cells is not simply a consequence of antigen overload but is actively induced by the immunosuppressive TME - specifically, by prostaglandin E2 (PGE2) produced by COX2-expressing TAMs, as discussed in detail in Section 3. Furthermore, scRNA-seq profiling of MSS CRC reveals enrichment of IL-17-producing T cells and a distinct multi-cellular immune hub at the tumor–luminal interface, associated with inflammatory myeloid-rich niches that may favor dysfunctional or exhausted T cell programs rather than robust Tpex maintenance (11). Collectively, these features establish that MSS CRC exhaustion is strongly shaped by non-cell-autonomous TME networks rather than by high neoantigen burden alone, and therefore is unlikely to be reversed by targeting any single inhibitory receptor in isolation. These T-cell-intrinsic programs operate within - and are amplified by - the multicellular immunosuppressive network described in the following sections, which provides the mechanistic context in which exhaustion is established and maintained (**Figure 1**).

## 3 Tumor-associated macrophages drive CD8+ T cell dysfunction and exhaustion in MSS CRC

### 3.1 TAM polarization and subtype distribution in MSS CRC

Tumor-associated macrophages (TAMs) are among the most abundant immune cells in the CRC TME and represent a central node in the immunosuppressive network that drives CD8+ T cell dysfunction and exhaustion (10, 11). TAMs are a highly plastic myeloid population whose functional polarization spans a spectrum from pro-inflammatory (M1-like) to immunosuppressive (M2-like) states, with the TME exerting dominant control over this polarization (27). While the M1/M2 dichotomy remains the classical framework, single-cell studies have refined TAM taxonomy into transcriptionally defined subsets (e.g., SPP1+, C1QC+, TREM2+), which are used here alongside the historical nomenclature. TAMs can orchestrate tumor immune evasion through cytokine secretion, metabolic reprogramming, and direct suppression of T cell function. In MSS CRC, TAMs are skewed toward an M2-like immunosuppressive polarization state, with significantly higher expression of COX1 and COX2 compared with MSI-H tumors - a difference that has direct mechanistic consequences for T cell function (9). Single-cell transcriptomic analyses have identified SPP1+ TAMs as a particularly immunosuppressive subpopulation that is specifically enriched in CRC, with SPP1 expression correlating with patient prognosis, TMB, MSI status, and immune checkpoint signaling (28). Spatial analyses across 14 independent CRC cohorts encompassing 2,550 patients have further demonstrated a consistent positive correlation and spatial co-localization between FAP+ cancer-associated fibroblasts (CAFs) and SPP1+ macrophages, suggesting that these two cell types cooperate to construct immune-exclusionary niches within the tumor stroma (29). The chemokine secretory profile of TAMs is itself a critical determinant of T cell trafficking: M2-polarized TAMs can preferentially secrete CCL2 and CCL22, which promote immunosuppressive cell recruitment, while suppressing the production of CXCL9 and CXCL10 that would otherwise attract cytotoxic T cells (30).

### 3.2 Molecular mechanisms by which TAMs drive CD8+ T cell dysfunction and exhaustion

#### 3.2.1 The COX2/PGE2–TIGIT axis: an MSS-specific mechanism of terminal exhaustion

The most mechanistically distinctive feature of TAM-driven exhaustion in MSS CRC is the COX2/PGE2–TIGIT axis. The immunosuppressive role of COX2-derived PGE2 in shaping the tumor immune microenvironment was established in a landmark study demonstrating that COX2 expression in cancer cells enables immune evasion by suppressing NK cell and T cell activity, and that COX2 inhibition restores anti-tumor immunity (31). In MSS CRC specifically, comparative single-cell, spatial, and bulk transcriptomic analyses have established that M2-like TAMs express high levels of COX1 and COX2, leading to elevated PGE2 production within the TME (9). PGE2 directly induces TIGIT upregulation on CD8+ T cells, driving them toward a PD-1+TIGIT+ terminally exhausted phenotype characterized by severely impaired cytokine secretion and cytolytic activity. Critically, this PD-1+TIGIT+ co-expression is largely absent in MSI-H tumors, where PD-1+ CD8+ T cells retain functional competence - establishing the COX2/PGE2–TIGIT axis as an MSS-specific mechanism of terminal exhaustion. Causal validation through macrophage depletion experiments confirmed that TAM ablation reduces TIGIT expression on CD8+ T cells and restores their functional activity, while also enhancing the efficacy of PD-L1 blockade. These findings provide a mechanistic rationale for combining COX2 inhibitors or EP2/EP4 receptor antagonists with checkpoint blockade to reverse TIGIT-mediated terminal exhaustion in MSS CRC.

#### 3.2.2 The SPP1–CD44 axis: suppression of Tc1 differentiation

A second, mechanistically distinct TAM-driven pathway operates through secreted phosphoprotein 1 (SPP1, also known as osteopontin). TAM-derived SPP1 engages CD44 on CD8+ T cells and impairs their differentiation toward the Tc1 effector fate, as evidenced by reduced TNF-α and IFN-γ production and disrupted activation of the LCK–ZAP70–LAT signaling cascade (28). *In vivo* validation using clodronate liposome-mediated macrophage depletion demonstrated reduced tumor growth and increased CD8+ T cell and Tc1 cell infiltration, effects that were partially reversed by recombinant SPP1 supplementation, confirming the specificity of this pathway. Notably, the SPP1–CD44 axis operates independently of the COX2/PGE2–TIGIT axis, acting on a distinct functional node: whereas PGE2 drives TIGIT-mediated terminal exhaustion, SPP1 blocks the upstream differentiation of functional Tc1 effectors. The two axes thus act in concert to suppress CD8+ T cell function through complementary mechanisms.

#### 3.2.3 The IDO1–kynurenine axis: metabolic immunosuppression

Indoleamine 2,3-dioxygenase 1 (IDO1) is significantly overexpressed in CRC tumors and correlates with TMB in TCGA-COAD/READ analyses (32). IDO1-expressing TAMs catabolize tryptophan to kynurenine, which activates the aryl hydrocarbon receptor (AhR) pathway in T cells, suppressing their proliferation and effector function. IDO1 knockdown impairs IFN-γ-mediated tolerance and increases apoptosis *in vitro*, effects that are rescued by exogenous kynurenine supplementation, confirming mechanistic specificity. *In vivo*, IDO1 inhibition combined with PD-1 blockade synergistically increases the infiltration of pro-inflammatory macrophages and CD8+ T cells through the JAK2–STAT3–IL-6 pathway, suggesting that IDO1 represents an important immunometabolic checkpoint in MSS CRC that can be exploited therapeutically.

### 3.3 TREM2+ TAMs and the SPP1+/mCAF spatial niche

Beyond their individual molecular mechanisms, TAMs contribute to CD8+ T cell exclusion, dysfunction, and exhaustion through their spatial organization within the TME. TREM2+ TAMs - a transcriptionally distinct subpopulation defined by expression of the triggering receptor expressed on myeloid cells 2 - are preferentially enriched in T cell-excluded zones and correlate with elevated expression of TIM-3, LAG-3, TIGIT, and PD-1 on tumor-infiltrating T cells (33). Mechanistically, TREM2+ TAMs suppress the formation of productive immune synapses between MHC+ macrophages and TCF1+ T cells, thereby impairing the maintenance of the Tpex pool. Preclinical studies demonstrate that combined blockade of TREM2, LAG-3, CTLA-4, and PD-1 achieves tumor clearance in over 70% of MMRp (MSS) mouse models, compared with near-complete clearance in MMRd models, by promoting interactions between MHC+C1Q+CXCL9+ macrophages and TCF1+PRF1+ T cells that generate durable immunological memory (33).

At the tissue scale, CRC liver metastases harbor a recurrent three-layered spatial ecosystem in which an outer belt of matrix CAFs (mCAFs) forms a stromal barrier, an adjacent band of terminally exhausted and stress-response T cells (Tex/TSTR) marks the arrested T cell compartment, and SPP1+ TAM aggregates accumulate along the inner tumor-facing boundary (34). This architecture is maintained by dominant intercellular communication axes - SPP1–CD44, FN1–CD44, and MIF/CXCL12 - that collectively promote T cell retention, metabolic stress, dysfunction, and exhaustion within the niche. Functional validation demonstrated that recombinant SPP1 and MIF impair CAR-T cell tumor clearance, establishing the causal role of this spatial ecosystem in therapeutic resistance. A three-gene prognostic signature derived from this niche (KLF2, ZBTB20, ARL4C) independently predicts recurrence and disease-free survival, providing a potential clinical tool for patient stratification.

## 4 Cancer-associated fibroblasts mediate CD8+ T cell exclusion and dysfunction

### 4.1 CAF heterogeneity and MSS CRC enrichment patterns

Cancer-associated fibroblasts (CAFs) constitute a major stromal component of the CRC TME and are increasingly recognized as active orchestrators of immune exclusion rather than passive bystanders. CAFs are a heterogeneous population of activated fibroblasts that arise from multiple cellular origins and adopt distinct functional states within the TME (35, 36). Single-cell transcriptomic studies across cancer types have resolved CAF heterogeneity into functionally specialized subpopulations with non-redundant roles in matrix remodeling, inflammatory signaling, and immune modulation (37). CAF nomenclature has evolved with single-cell resolution; the myCAF/iCAF/mCAF (and apCAF-related) descriptors used here correspond to, and partly supersede, earlier activation-state and marker-based designations (36). These include myofibroblastic CAFs (myCAFs, αSMA+) that remodel the extracellular matrix (ECM); inflammatory CAFs (iCAFs, IL-6+/IL-1R1+) that propagate inflammatory and immunosuppressive signals; and matrix CAFs (mCAFs) that construct physical barriers to T cell infiltration. In MSS CRC, which is enriched for the consensus molecular subtype 4 (CMS4) - a mesenchymal subtype characterized by TGF-β pathway activation and stromal invasion - fibroblast subpopulations are expanded and correlate with immune exclusion and adverse prognosis. The IL-1 receptor 1 (IL1R1) is expressed at significantly higher levels in CMS4 patients than in CMS1–3, implicating iCAFs as a functionally important subpopulation in this MSS-enriched context (38). FAP+ fibroblasts, a canonical marker of immune-exclusionary CAFs, consistently co-localize spatially with SPP1+ macrophages across independent CRC cohorts within desmoplastic immune-excluded regions and are associated with reduced lymphocyte infiltration (29).

### 4.2 TGF-β as the upstream driver of CAF-mediated T cell exclusion

TGF-β signaling in the tumor stroma is a major upstream mechanism linking CAF activation to T cell exclusion in MSS CRC. Two landmark studies published simultaneously in Nature established the causal role of this pathway. Mariathasan et al., in metastatic urothelial cancer, demonstrated that TGF-β signaling activates peri-tumoral fibroblasts, physically confining CD8+ T cells to the tumor stroma and invasive margin while excluding them from the tumor parenchyma; combined TGF-β receptor blockade and PD-L1 inhibition reversed T cell exclusion and produced complete responses in preclinical models (39). Complementarily, Tauriello et al. showed in a genetically reconstituted CRC metastasis model that high stromal TGF-β activity drives T cell exclusion from the tumor core, and that TGF-β blockade combined with PD-L1 therapy restored T cell infiltration and achieved tumor regression (39). These findings are particularly relevant to MSS CRC, where TGF-β pathway activation is substantially higher than in MSI-H tumors, and where TGF-β-driven fibroblast activation may converge with downstream mechanisms - including THBS2-mediated CXCL9/10 suppression - to enforce immune exclusion.

### 4.3 The THBS2–CXCL9/10–CXCR3 axis: fibrosis-driven immune exclusion

Within the fibrotic TME of MSS CRC, the glycoprotein thrombospondin-2 (THBS2) has been identified as a key effector of CAF-mediated immune exclusion. THBS2 is highly expressed by mCAFs at the tumor invasive front and acts by suppressing the production of the T cell-attracting chemokines CXCL9 and CXCL10 by dendritic cells and macrophages, thereby preventing CXCR3+ CD8+ T cells from being recruited to the tumor margin (40). In orthotopic CRC models, systemic or fibroblast-specific deletion of Thbs2 disrupts the immune-exclusionary barrier and significantly increases intra-tumoral CD8+ T cell density. Importantly, however, THBS2 deletion alone does not restore T cell function - the infiltrating CD8+ T cells remain exhausted - suggesting that physical exclusion and functional exhaustion are parallel but distinct barriers that likely both need to be addressed for therapeutic benefit. This finding supports combining THBS2 targeting with checkpoint blockade to achieve more complete immune reprogramming in fibrotic MSS CRC.

### 4.4 CAFs directly impair CD8+ T cell effector function

Beyond orchestrating T cell exclusion, CAFs also directly impair the function of T cells that do succeed in infiltrating the tumor through at least two distinct mechanisms.

#### 4.4.1 Senescent CAF–CD36–lipid peroxidation axis

Single-cell transcriptomic analyses of CRC tissue have identified a senescent fibroblast subpopulation that is absent in normal adjacent mucosa and that exhibits enhanced intercellular communication with CD8+ T cells (41). Co-culture experiments demonstrate that senescent CAFs significantly impair the cytotoxic function of CD8+ T cells against CRC organoids, reducing granzyme B (GZMB+) and IFN-γ+ T cell fractions while increasing organoid survival. The mechanistic basis for this effect is lipid transfer: senescent CAFs transfer lipids to CD8+ T cells via a CD36-dependent mechanism, inducing lipid peroxidation and consequent functional exhaustion, as confirmed by direct lipid-tracking experiments using fluorescent fatty acid analogues. *In vivo*, senolytic agents (compounds that selectively eliminate senescent cells) suppress CRC progression in AOM/DSS mouse models, and high CD36 expression in TCGA-COAD correlates with poor prognosis, nominating CD36 as both a functional mediator and a prognostic biomarker.

#### 4.4.2 MFAP2–ITGB8–ERK1/2–CYP27A1–LXRβ metabolic cascade

A second CAF-to-cancer-cell-to-T-cell signaling relay has been delineated in which CAF-derived microfibrillar-associated protein 2 (MFAP2) engages integrin β8 (ITGB8) on CRC cells, activating FAK–ERK1/2 signaling, which drives ETS2-mediated transcriptional upregulation of CYP27A1 - a cytochrome P450 enzyme that generates oxysterols activating the nuclear receptor LXRβ - ultimately suppressing CD8+ T cell function (42). This cascade is unique in its cross-cellular architecture: CAF-derived MFAP2 acts on cancer cells, which in turn suppress T cells through a lipid metabolic intermediary, illustrating how CAFs can exert immunosuppressive effects through indirect, multi-step signaling relays.

### 4.5 IL1R1+ iCAFs: inflammatory amplifiers of the immunosuppressive network

The inflammatory CAF subpopulation defined by IL-1 receptor 1 (IL1R1) expression plays a distinct amplifying role in the immunosuppressive network of MSS CRC. IL-1β stimulation of IL1R1+ iCAFs induces the expression of 829 differentially expressed genes - compared with only 46 in normal fibroblasts - underscoring the exquisite sensitivity of this subpopulation to inflammatory signals (38). Mechanistically, activated iCAFs secrete MCP-1/CCL2 to recruit and promote M2 polarization of macrophages, simultaneously suppressing T cell proliferation. Fibroblast-specific IL1R1 deletion in murine CRC models improves survival, reduces tumor growth, and decreases Th17 cell infiltration.

#### 4.5.1 CAF-derived WNT2 and NNMT as emerging immunosuppressive mediators

Beyond the established CAF axes, two additional CAF-derived factors have been identified as immunosuppressive mediators in CRC. CAF-secreted WNT2 suppresses dendritic cell-mediated anti-tumor immunity, and its targeting restores DC function and enhances T cell responses (43). More recently, nicotinamide N-methyltransferase (NNMT) in CAFs has been identified as a metabolic immunosuppressive enzyme: NNMT-induced H3K27me3 hypomethylation drives complement secretion from CAFs, which recruits MDSCs to the tumor and suppresses CD8+ T cell activation; NNMT inhibition reduces MDSC recruitment and restores CD8+ T cell activity across ovarian, breast, and colon syngeneic models, with NNMTi plus checkpoint blockade combinations validated in ovarian, breast, and colon (MC38) syngeneic models, establishing a new metabolic axis linking CAF epigenetic reprogramming to myeloid-mediated T cell suppression (44). Spatial transcriptomic analysis of rectal cancer specimens following neoadjuvant chemotherapy (NAC) has revealed that NAC markedly reshapes CAF subset composition: pro-immune CAF subsets (CAF_PI16, CAF_SLIT2) expand in complete responders and activate CD8+ T cell recruitment through CXCL12 and SLIT2 signaling, while the immunosuppressive CAF_FAP subset - which promotes epithelial-mesenchymal transition via MIR4435-2HG induction - persists in non-responders and correlates with poor outcomes (45). This CAF subset heterogeneity in response to chemotherapy provides a mechanistic basis for understanding why NAC response is variable in pMMR/MSS CRC and suggests that co-targeting immunosuppressive CAF subsets (particularly CAF_FAP) alongside chemotherapy may improve outcomes.

#### 4.5.2 CAF heterogeneity caveat

Not all CAF subsets are immunosuppressive: a CCL19+ CAF subset in CRC liver metastases promotes TLS formation and counterbalances the immunosuppressive CAF majority, illustrating that the CAF compartment harbors immune-supportive subpopulations alongside the dominant suppressive ones (46). These examples extend the CAF axis from physical exclusion to broader regulation of CD8+ T cell access, activation, and myeloid-mediated suppression.

IL1R1+ iCAFs thereby function as inflammatory amplifiers that bridge CAF activation, TAM polarization, and T cell suppression, forming a positive feedback circuit with M2-TAMs that consolidates the immunosuppressive architecture of MSS CRC.

## 5 Regulatory T cells suppress CD8+ T cell anti-tumor function

### 5.1 The CRC paradox: context-dependent Treg function

The relationship between regulatory T cells (Tregs) and tumor immunity in CRC is paradoxical and has long been a source of controversy. Tregs are a specialized CD4+ T cell lineage defined by FoxP3 expression and dedicated to maintaining immune homeostasis and self-tolerance; their core biology and suppressive mechanisms have been extensively characterized (47). In the tumor context, Tregs are generally viewed as pro-tumorigenic through their suppression of anti-tumor effector T cell responses (48). A meta-analysis of 76 studies encompassing 15,512 cancer patients established that high FoxP3+ Treg infiltration is associated with improved overall survival in CRC - in striking contrast to the negative prognostic effect observed in the majority of other solid tumors, including cervical, renal, and breast cancers (49). This CRC-specific paradox, in which Tregs appear to exert context-dependent pro- and anti-tumor effects, has now been mechanistically resolved by the discovery of functionally distinct Treg subpopulations with opposing roles in MSS CRC. In MSS CRC, FoxP3+ Tregs are significantly enriched relative to MSI-H tumors and inversely correlate with CD8+ T cell density, consistent with their immunosuppressive function (2). Tumor-infiltrating Tregs exhibit markedly higher immunosuppressive activity than their peripheral blood counterparts, with 63.6 ± 16.0% expressing CCR8 - a chemokine receptor that marks the most potently suppressive intra-tumoral Treg fraction (50).

### 5.2 Functional dichotomy of Treg subpopulations identified in MSS CRC

A landmark study using an orthotopic, MSS CRC, integrated with single-cell multi-omics (scRNA-seq and ATAC-seq), resolved the long-standing CRC Treg paradox into two functionally antagonistic intratumoral Treg subsets defined by their differential capacity to express IL-10 (51). The IL-10− subset is enriched within the tumor, expands as disease progresses, and is pro-tumorigenic: its selective depletion elicits pronounced tumor regression. The IL-10+ subset, by contrast, predominates in normal colonic mucosa and exerts an unexpected tumor-restraining function: its selective ablation accelerates tumor growth by releasing CD4+ effector cells whose IL-17 production directly drives tumor-cell proliferation. This IL-17 bridge is consistent with the well-documented pro-tumorigenic role of the IL-23/IL-17 axis in CRC (52, 53), which both promotes tumor-cell proliferation and suppresses CD8+ cytotoxic T cell and regulatory T cell recruitment by repressing CXCL9/CXCL10 (52). The favorable prognosis associated with high bulk FoxP3+ Treg infiltration in CRC (49) therefore likely reflects the combined contribution of the IL-10+ anti-tumor fraction, which cannot be distinguished from the pro-tumorigenic IL-10− population in bulk FoxP3+ analyses. Validation in human CRC confirmed that IL-10+ and IL-10− Treg abundances correlate with favorable and unfavorable prognosis, respectively, and transcriptionally similar subsets were observed across other human barrier-tissue tumors (51), suggesting cross-cancer conservation of this dichotomy. These findings argue for therapeutic strategies that preserve IL-10+ Tregs while selectively depleting the CCR8+ IL-10− fraction, thereby relieving CD8+ T cell suppression without removing a tumor-restraining regulatory population.

### 5.3 CCR8+ Tregs: the pro-tumorigenic fraction and its therapeutic targeting

CCR8 expression identifies the pro-tumorigenic IL-10− Treg fraction within the MSS CRC TME. CCR8+ tumor-infiltrating Tregs express high levels of activation markers and exhibit potent immunosuppressive activity, and their abundance correlates with poor patient survival (50). Anti-CCR8 monoclonal antibody treatment in CT26 subcutaneous tumor models effectively reduces intra-tumoral Treg density and restores CD4+ and CD8+ T cell anti-tumor immunity. The co-expression of CCR8 and CTLA-4 on tumor-infiltrating Tregs has been exploited to develop a bispecific antibody (2MW4691) that simultaneously depletes CCR8+ Tregs and relieves CTLA-4-mediated suppression of CD8+ T cells - a “two-birds-one-stone” strategy that achieves greater anti-tumor efficacy than either target alone (54). Because IL-10− Tregs preferentially express CCR8, CCR8 targeting may preferentially affect this pro-tumorigenic fraction, potentially avoiding the paradoxical tumor-promoting effect that would result from indiscriminate Treg depletion.

### 5.4 Mechanisms of Treg-mediated CD8+ T cell suppression

Tregs suppress CD8+ T cell function through multiple, partially redundant mechanisms. Direct contact-dependent suppression occurs primarily through CTLA-4-mediated competitive sequestration of CD80/CD86 on antigen-presenting cells, depriving CD8+ T cells of co-stimulatory signals required for activation and survival; cytotoxic pathways such as granzyme/perforin and Fas/FasL have also been implicated in responder-cell death in some contexts. Cytokine-mediated suppression is effected by IL-10, TGF-β, and IL-35, which collectively inhibit CD8+ T cell effector function, proliferation, and cytokine production. Metabolic competition represents a third mechanism: Tregs constitutively express high levels of CD25 (IL-2Rα) and thereby competitively consume IL-2 from the TME, depriving CD8+ T cells of this essential survival and proliferative signal. Indirectly, Tregs promote M2 polarization of TAMs through IL-10 and TGF-β secretion, amplifying the immunosuppressive network beyond their direct effects on T cells. The convergence of these mechanisms with MDSC-mediated arginine depletion and ROS production creates a state of compounded metabolic and functional deprivation for CD8+ T cells in MSS CRC. Treg-driven suppression therefore primarily contributes to the dysfunction layer of the triad, with indirect amplification of exhaustion through sustained inhibitory signaling rather than direct enforcement of physical exclusion.

## 6 Myeloid-derived suppressor cells impair CD8+ T cell function

### 6.1 MDSC expansion and distribution in MSS CRC

Myeloid-derived suppressor cells (MDSCs) - a heterogeneous population of immature myeloid cells comprising polymorphonuclear MDSCs (PMN-MDSCs) and monocytic MDSCs (MO-MDSCs) - were first characterized as a pathologically activated myeloid population that expands in cancer and other chronic inflammatory conditions to suppress T cell responses (55). International consensus has standardized their nomenclature and classification criteria, distinguishing PMN-MDSCs from granulocytes and MO-MDSCs from monocytes based on functional immunosuppressive activity (56). This nomenclature supersedes earlier organ- and surface-marker-based labels (e.g., CD15+/CD11b+ granulocytic; CD14+/CD11b+ monocytic cells) and is the classification followed throughout this review. MDSCs are generally enriched in MSS/pMMR immune-cold CRC relative to MSI-H immune-inflamed tumors (2). This expansion reflects both increased myelopoiesis driven by tumor-derived factors and impaired terminal differentiation of myeloid progenitors within the TME. In MSS CRC, MDSC enrichment is accompanied by a reciprocal reduction in NK cells and dendritic cells, creating a myeloid-dominated immunosuppressive milieu that is particularly refractory to checkpoint blockade (57). KRAS mutations, common in CRC and frequently present in MSS disease, drive MDSC recruitment through the IRF2–CXCL3–CXCR2 axis, linking a major oncogenic driver of MSS CRC to its immunosuppressive microenvironment (58). Single-cell and spatial proteomic analyses have further established that immunosuppressive myeloid states - encompassing both MDSCs and M2-like TAMs - form an integrated cellular barrier to checkpoint inhibitor efficacy that is a defining feature of MSS CRC (59).

### 6.2 Multi-mechanistic suppression of CD8+ T cells by MDSCs

MDSCs are a heterogeneous population whose suppressive mechanisms have been comprehensively reviewed (60). The m6A RNA modification pathway has emerged as an additional regulatory layer controlling MDSC function: targeting YTHDF1, an m6A reader protein, augments anti-tumor immunity and enhances anti-PD-1 efficacy in CRC by modulating myeloid cell differentiation and function (61), complementing the Mettl3-Smad3 axis described below. In MSS CRC, MDSCs impair CD8+ T cell function through at least three mechanistically distinct categories of immunosuppression.

#### 6.2.1 Amino acid depletion

MDSCs constitutively express high levels of arginase 1 (ARG-1), which depletes L-arginine from the TME. Arginine deprivation downregulates the TCR-ζ chain on CD8+ T cells, impairing TCR signaling and abrogating T cell proliferation and IFN-γ production. Concurrently, inducible nitric oxide synthase (iNOS) in MO-MDSCs generates nitric oxide (NO), which nitrosylates the TCR, disrupts antigen-specific signaling, and nitrosates CCL2 to impair chemokine-driven T cell recruitment. Together, ARG-1 and iNOS impose a state of metabolic starvation on CD8+ T cells that mimics the consequences of TCR signaling failure.

#### 6.2.2 Oxidative stress

MDSCs produce reactive oxygen species (ROS) and peroxynitrite, which nitrate and oxidize the TCR and its co-receptors, rendering T cells antigen-unresponsive. Peroxynitrite-mediated nitration of the TCR is particularly damaging as it selectively abolishes antigen-specific responses while preserving non-specific T cell activation, effectively uncoupling T cell function from tumor antigen recognition.

#### 6.2.3 PD-L1-mediated direct exhaustion

Hypoxia within the MSS CRC TME drives HIF-1α-dependent transcriptional upregulation of PD-L1 on MDSCs - a direct regulatory relationship that has been experimentally validated: HIF-1α is a direct transcriptional activator of the PD-L1 promoter, and hypoxia selectively and rapidly upregulates PD-L1 on MDSCs (but not other inhibitory receptors), with MDSC PD-L1 levels at tumor sites substantially exceeding those in peripheral lymphoid organs (62). MDSC-expressed PD-L1 engages PD-1 on CD8+ T cells, directly triggering exhaustion signaling and functionally mimicking the PD-L1/PD-1 axis on tumor cells. Importantly, PD-L1 blockade under hypoxic conditions restores MDSC-mediated T cell activation, establishing that hypoxia–HIF-1α–PD-L1 on MDSCs is a relevant therapeutic target in the hypoxic cores of MSS CRC tumors.

### 6.3 Epigenetic impairment of MO-MDSC maturation: the Smad3–m6A axis

A newly characterized mechanism of MDSC dysfunction in CRC involves the epigenetic impairment of monocytic MDSC (MO-MDSC) terminal differentiation. Smad3 - a canonical TGF-β signal transducer - is significantly downregulated in MDSCs from CRC patients and tumor-bearing mouse models, and this downregulation blocks MO-MDSC maturation into immunostimulatory macrophages and dendritic cells (63). Myeloid-specific Smad3 overexpression restores MO-MDSC differentiation into mature antigen-presenting cells, upregulates MHC class II expression, reduces immunosuppressive molecule production, and attenuates tumor progression. The mechanistic basis for Smad3 downregulation is post-transcriptional: Mettl3-mediated N6-methyladenosine (m6A) modification of the Smad3 mRNA 3′-UTR destabilizes the transcript, linking RNA epigenetics to MDSC dysfunction. Paradoxically, TGF-β1 - which signals through Smad3 in canonical pathways - instead promotes MO-MDSC expansion via the non-canonical PI3K/AKT pathway in this context, while Smad3 overexpression counteracts this pro-MDSC effect. Clinically, plasma TGF-β1 levels correlate with the MO-MDSC/PMN-MDSC ratio across cancer types, highlighting its potential as a biomarker for MDSC-dominated immunosuppression in MSS CRC.

### 6.4 MDSC crosstalk with Tregs, TAMs, and CAFs

MDSCs do not operate in isolation but engage in extensive bidirectional crosstalk with the other immunosuppressive cell types in the MSS CRC TME. MDSC-derived TGF-β and IL-10 promote Treg differentiation and expansion, while Tregs in turn enhance MDSC suppressive function through IL-10 feedback, creating a self-reinforcing immunosuppressive loop. MDSCs promote M2 polarization of TAMs through IL-10 and prostaglandin secretion, amplifying the COX2/PGE2–TIGIT and SPP1–CD44 axes described in Section 3. CAF-derived VEGF and IL-6 promote MDSC expansion and functional activation, linking the structural stromal compartment to the myeloid immunosuppressive network. This multi-cellular positive feedback architecture ensures that even partial depletion of one MDSC-dependent axis can be compensated by others - a key reason why single-agent targeting strategies have generally produced limited or inconsistent efficacy in MSS CRC and why multi-targeted approaches are likely required. MDSCs thus act predominantly on the dysfunction and metabolic-deprivation axes of the triad, with their direct contribution to exhaustion restricted to PD-L1 signaling under hypoxia.

## 7 The integrated immunosuppressive network: cellular crosstalk and spatial architecture

### 7.1 Bidirectional axes linking the four cellular compartments

The immunosuppressive network of MSS CRC is not the sum of four independent inhibitory axes but an integrated system sustained by extensive bidirectional crosstalk between TAMs, CAFs, Tregs, and MDSCs. The TAM–CAF axis supports a model in which IL1R1+ iCAFs and M2-TAMs engage in reciprocal activation: iCAFs secrete CCL2 and IL-6 to recruit monocytes and promote their M2 polarization, while macrophages contribute IL-1β that further activates iCAFs, together reinforcing the fibrotic and immunosuppressive features of the TME (38). Spatial co-localization of FAP+ CAFs and SPP1+ macrophages, documented across 14 independent cohorts, is the morphological manifestation of this reciprocal activation loop - a fibroblast–macrophage unit that constructs the structural scaffolding of immune-excluded tumor territory (29). The Treg–MDSC axis operates through parallel IL-10/TGF-β circuits: MDSC-derived TGF-β induces Treg differentiation, while Treg-derived IL-10 enhances MDSC suppressive potency. Mechanistically these populations act on distinct nutrient/cytokine axes - MDSCs impose arginine/nitrogen stress through ARG-1 and iNOS, whereas Tregs deprive effector T cells of IL-2 through high CD25 expression - together creating complementary metabolic and cytokine deprivation that is more limiting than either population alone (**Table 1**).

**Table 1:** Key cellular subpopulations driving CD8+ T cell dysfunction in MSS CRC.

Cell subpopulation	Defining markers	Primary molecular mechanism	Main triad axis	Key references
M2-like TAMs (general)	M2-like; COX1/2-high in MSS vs MSI-H	Elevated PGE2 production; CCL2/CCL22 secretion with suppressed CXCL9/10	Exhaustion (terminal); Exclusion (chemokine)	(9, 30)
SPP1+ TAMs	SPP1+ (osteopontin-high)	SPP1–CD44 → impaired Tc1 differentiation, blunted LCK/ZAP70/LAT; spatial co-localization with FAP+ CAFs	Dysfunction	(28, 29, 34)
TREM2+ TAMs	TREM2+	Suppress productive synapse between MHC+ macrophages and TCF1+ Tpex; impair Tpex maintenance	Exhaustion (Tpex depletion)	(33)
IDO1+ TAMs	IDO1+	Tryptophan → kynurenine → AhR activation in T cells (JAK2/STAT3/IL-6 associated with response to IDO1 inhibition + PD-1, not the suppressive mechanism itself)	Dysfunction (metabolic)	(32)
MS4A4A+ TAMs	MS4A4A+	Immunosuppressive surface marker; anti-MS4A4A reprograms toward stimulatory phenotype	Dysfunction	(77)
FAP+/THBS2-high stromal CAF programs	FAP+, THBS2-high at invasive front; overlapping with mCAF/myCAF programs (αSMA marks the myCAF subset)	THBS2 suppresses dendritic-cell/macrophage CXCL9/10; ECM barrier; co-localized with SPP1+ TAMs	Exclusion (primary)	(29, 34, 40)
IL1R1+ inflammatory CAFs (iCAFs)	IL1R1+ inflammatory CAF program; IL-1β-responsive, CCL2/IL-6-producing	IL-1β → CCL2/IL-6 → M2-TAM polarization; T cell proliferation suppression	Dysfunction (network amplifier)	(38)
Senescent CAFs	SASP+; CD36-ligand donor	Lipid transfer to CD8+ T cells via CD36 → lipid peroxidation → functional exhaustion	Dysfunction/exhaustion-like metabolic impairment	(41)
MFAP2+ CAFs	MFAP2-secreting	MFAP2 → ITGB8 on tumor cells → FAK-ERK-ETS2 → CYP27A1 → LXRβ oxysterols suppress CD8+ T cells	Dysfunction (indirect)	(42)
NNMT+ CAFs	NNMT-high; H3K27me3-hypomethylated	Complement secretion → MDSC recruitment; validated in ovarian, breast, and colon (MC38) models — not specifically MSS CRC	Dysfunction (via MDSC)	(44)
Persistent CAF_FAP (post-NAC)	FAP+ surviving neoadjuvant chemotherapy	EMT via MIR4435-2HG; associated with non-response to NAC in rectal cancer (no direct T cell exclusion mechanism demonstrated)	NAC resistance/EMT-associated immunosuppressive CAF	(45)
CCL19+ CAFs (immune-supportive, in CRC liver metastases)	CCL19+	Identified in CRC liver metastases; promote TLS formation and anti-tumor IgG responses — counterbalance suppressive CAF majority	Immune-supportive (anti-suppression)	(46)
IL-10−/CCR8+ Tregs (pro-tumor)	FoxP3+, CCR8+, IL-10−	Intratumoral; CCR8+ activation phenotype; CTLA-4-mediated CD80/86 sequestration; IL-2 consumption via CD25; pro-tumor suppressive Treg program	Dysfunction (active suppression)	(50, 51, 54)
IL-10+ Tregs (anti-tumor)	FoxP3+, IL-10+	Predominantly in normal mucosa; restrain pro-tumorigenic IL-17 production by CD4+ effectors	Tumor-restraining (preserve)	(51)
PMN-MDSCs	CD11b+Ly6G+ (mouse)/CD15+ (human)	ARG-1 → L-arginine depletion → TCR-ζ downregulation; ROS/peroxynitrite → TCR nitration (per manuscript these mechanisms are general to MDSCs; PMN-enriched per consensus, not exclusively PMN)	Dysfunction (metabolic + TCR)	(55, 56, 60)
MO-MDSCs	CD11b+Ly6C+ (mouse)/CD14+ (human)	iNOS → NO-mediated TCR nitrosylation (MO-MDSC-specific); HIF-1α-driven PD-L1 on MDSCs under hypoxia (not MO-restricted in manuscript); Mettl3-m6A → Smad3 instability blocks MO-MDSC maturation	Dysfunction; exhaustion signal from hypoxic MDSC PD-L1	(61–63)

Cellular subpopulations of TAMs, CAFs, Tregs, and MDSCs implicated in CD8+ T cell exclusion, dysfunction, and exhaustion in MSS colorectal cancer. The “Main triad axis” column indicates which of the three title axes the subpopulation principally drives; where evidence is mainly clinical or treatment-resistance-related rather than direct triad mechanism, this is noted explicitly. TAM, tumor-associated macrophage; CAF, cancer-associated fibroblast; Treg, regulatory T cell; MDSC, myeloid-derived suppressor cell; SASP, senescence-associated secretory phenotype; TLS, tertiary lymphoid structure; NAC, neoadjuvant chemotherapy.

### 7.2 A four-layer model of immunosuppression

The coordinated activity of the four cellular axes can be conceptualized as operating through four hierarchically organized layers of immunosuppression that sequentially prevent, impair, and eliminate effective CD8+ T cell responses.

#### 7.2.1 The first layer - physical exclusion

Physical exclusion is constructed by mCAF-derived THBS2, which suppresses CXCL9/10 production by dendritic cells and macrophages, depriving CXCR3+ CD8+ T cells of the chemokine gradient required for recruitment to the tumor invasive front (40). TGF-β-activated peri-tumoral fibroblasts reinforce this barrier by depositing a dense ECM that physically impedes T cell migration (39, 64). In melanoma, Wnt/β-catenin signaling in tumor cells has been established as an upstream driver of T cell exclusion through suppression of CCL4-dependent dendritic cell recruitment, providing a paradigm for oncogene-driven immune exclusion that may converge with stromal mechanisms on the same immune-exclusionary outcome (65).

#### 7.2.2 The second layer - functional impairment

Functional impairment acts on CD8+ T cells that succeed in penetrating the physical barrier. TAM-derived PGE2 upregulates TIGIT on infiltrating CD8+ T cells (9), while SPP1 blocks Tc1 differentiation (28), senescent CAF-mediated lipid transfer induces lipid peroxidation (41), and MFAP2-driven LXRβ signaling in cancer cells further suppresses T cell effector function (42).

#### 7.2.3 The third layer - metabolic deprivation

Metabolic deprivation is imposed primarily by MDSCs (ARG-1/iNOS-mediated arginine and nitrogen depletion, ROS-mediated TCR nitration) and IDO1-expressing TAMs (tryptophan catabolism to immunosuppressive kynurenine) (32), creating a metabolically hostile environment in which even structurally intact CD8+ T cells cannot sustain effector function.

#### 7.2.4 The fourth layer - active suppression

**Figure 2:**
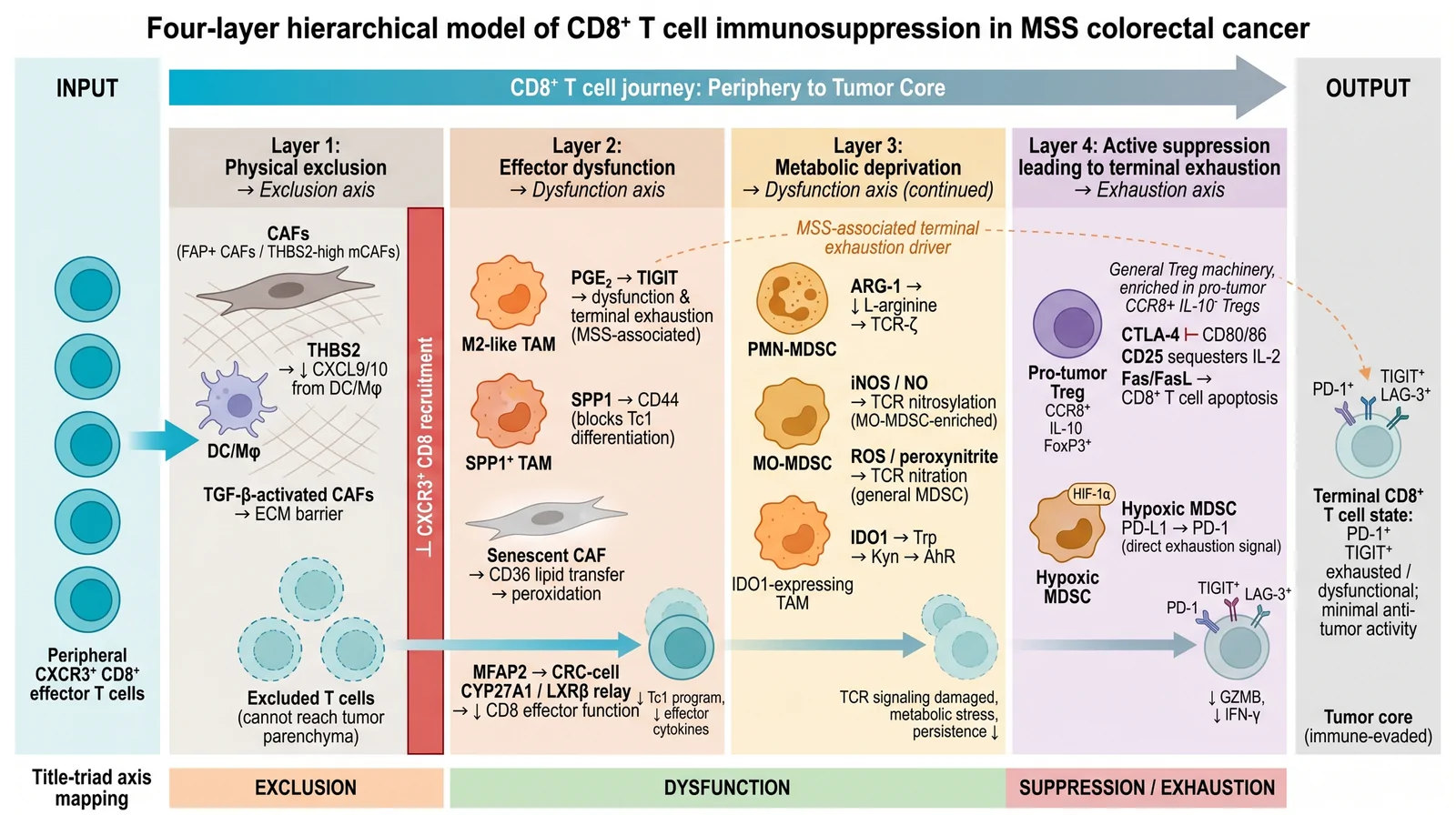
Four-layer hierarchical model of sequential immunosuppression driving CD8^+^ T cell exclusion, dysfunction, and terminal exhaustion in microsatellite-stable colorectal cancer.

Active suppression is mediated by Tregs, which deplete IL-2 and can induce CD8+ T cell apoptosis via Fas/FasL, while MDSC-expressed PD-L1 provides an additional direct exhaustion signal (62). This layered architecture means that CD8+ T cells face sequential, compounding barriers: exclusion limits entry, functional impairment weakens effector activity, metabolic deprivation limits persistence, and active suppression reinforces exhaustion (**Figure 2**).

### 7.3 Spatial architecture: the three-layer immune-exclusionary niche

**Figure 3:**
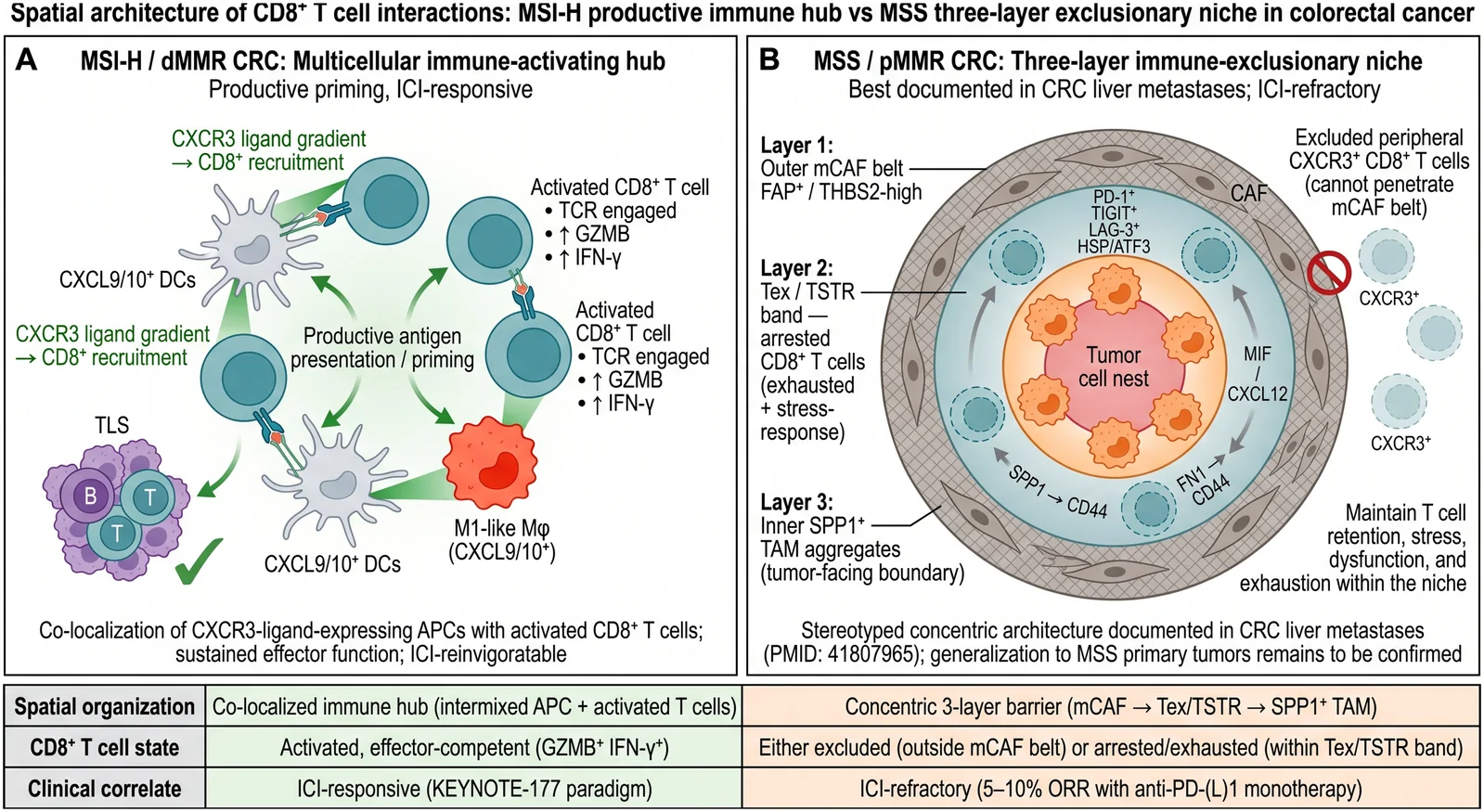
Contrasting spatial architectures of CD8⁺ T cell interactions in colorectal cancer: the MSI-H multicellular immune hub versus the MSS three-layer exclusionary niche. **(A)** In MSI-H/dMMR CRC, CXCL9/10^+^ dendritic cells and M1-like macrophages create CXCR3 ligand gradients to recruit CD8^+^ T cells into a co-localized multicellular immune-activating hub built around productive antigen presentation. This configuration maintains effector-competent activated CD8^+^ T cells (GZMB^+^ IFN-γ^+^), is associated with tertiary lymphoid structures, and confers ICI responsiveness. **(B)** MSS/pMMR CRC features a stereotyped three-layer immune-exclusionary niche that insulates tumor cells from T cell-mediated killing. This structure is well characterized in CRC liver metastases, yet whether it applies to primary MSS tumors remains unconfirmed. The outer FAP^+^/THBS2-high mCAF belt blocks infiltration of peripheral CXCR3^+^ CD8^+^ T cells; the intermediate layer consists of functionally arrested terminally exhausted stress-response T cells (Tex/TSTR; PD-1^+^ TIGIT^+^ LAG-3^+^); inner SPP1^+^ TAM aggregates produce intercellular communication signals (SPP1–CD44, FN1–CD44, MIF, CXCL12) that drive T cell retention, stress response and exhaustion. This concentric barrier renders tumors refractory to ICI therapy. The bottom table summarizes distinctions in spatial organization, CD8^+^ T cell phenotype and clinical correlates for the two CRC subtypes.

The multi-cellular immunosuppressive network is not randomly distributed but is spatially organized into a stereotyped three-layer structure, best documented in CRC liver metastases using spatial transcriptomics and multiplex imaging - and whose generalization to non-metastatic MSS primary tumors remains to be confirmed (34). CODEX multiplexed imaging of CRC tissue has demonstrated that the spatial relationships between immune and stromal cells encode functional information about the immunosuppressive state of the TME, with the coupling and fragmentation of immune and stromal cellular neighborhoods predicting clinical outcome (66). An outer belt of mCAFs forms the structural boundary of the immune-excluded territory. Adjacent to this mCAF belt is a band of terminally exhausted and stress-response T cells (Tex/TSTR) - CD8+ T cells that have entered the tumor but are functionally arrested by the surrounding immunosuppressive signals. SPP1+ TAM aggregates accumulate along the inner tumor-facing boundary and produce the intercellular communication signals (SPP1–CD44, FN1–CD44, MIF, CXCL12) that promote T cell retention and stress responses. This spatial organization is mechanistically distinct from the multi-cellular immune hub architecture described in MSI-H CRC, where CXCR3 ligand-expressing cells co-localize with activated T cells to form productive immune activation niches (11). A comprehensive framework for classifying TME states based on transcriptomic signatures of immune and stromal populations has been proposed, providing a conceptual basis for predicting immunotherapy responsiveness across cancer types (67, 68). TLS density and localization have been validated as independent prognostic biomarkers in CRC liver metastases, with high TLS density associating with favorable molecular subtypes and improved clinical outcomes (24, 69). In contrast to the exclusionary mCAF–SPP1+ TAM–Tex/TSTR niche, TLS-rich regions may represent spatially distinct immune-supportive niches. The spatiotemporal dynamics of the CRC immune landscape - including the cellular transitions that distinguish immunotherapy responders from non-responders - have been decoded by single-cell analyses that track the evolution of T cell exhaustion states in relation to the surrounding immunosuppressive network (25). In MSS CRC, the equivalent spatial unit is an immune-exclusionary niche rather than an immune-activating one - a fundamental architectural difference that explains the divergent clinical responses to checkpoint blockade between the two subtypes (**Figure 3**).

### 7.4 Summary: MSS CRC immune resistance as a network phenomenon

The integrated four-layer, spatially organized immunosuppressive network of MSS CRC represents a fundamentally different challenge from the predominantly antigen-driven immune dysfunction of MSI-H disease. Three features distinguish this network-driven immune resistance and have direct therapeutic implications. First, the MSS-specific PD-1+TIGIT+ terminal exhaustion phenotype is actively maintained by ongoing TAM-derived PGE2 signaling, meaning that checkpoint blockade alone cannot prevent its re-establishment even if T cell function is transiently restored (9). Second, the Tpex pool in MSS CRC is continuously depleted by the cellular interaction network - TAM–SPP1, CAF–TGF-β, and Treg–MDSC circuits drive Tpex-to-Ttex conversion faster than peripheral replenishment can compensate - meaning that the substrate for checkpoint-mediated reinvigoration is progressively exhausted. Third, the spatial organization of the immune-exclusionary niche physically separates the therapeutic target (CD8+ T cells) from the replenishment pool, further limiting the reach of systemic immunotherapy. Overcoming MSS CRC immune resistance therefore requires strategies that simultaneously dismantle the physical barrier (anti-TGF-β/THBS2), reprogram the myeloid compartment (anti-TREM2/COX2/IDO1), selectively deplete pro-tumorigenic Tregs (anti-CCR8), and neutralize the metabolic suppression layer (anti-MDSC/ARG-1) - a multi-pronged approach that is the focus of the following section.

## 8 Therapeutic strategies to overcome CD8+ T cell exclusion, dysfunction, and exhaustion in MSS CRC

### 8.1 Targeting the TAM axis

#### 8.1.1 COX2/PGE2–TIGIT blockade

The mechanistic centrality of the COX2/PGE2–TIGIT axis in MSS CRC terminal exhaustion provides a compelling rationale for combining COX2 inhibitors or EP2/EP4 prostaglandin receptor antagonists with checkpoint blockade. The development of immune checkpoint inhibitors as a therapeutic modality was itself founded on the principle that releasing T cells from inhibitory signals can restore durable anti-tumor immunity (70), and the subsequent demonstration that combination checkpoint strategies can overcome resistance to single-agent PD-1 blockade has motivated the search for rational combination partners in MSS CRC (71). Preclinical studies demonstrate that pharmacological suppression of PGE2 signaling reduces TIGIT expression on CD8+ T cells and restores their functional activity, sensitizing tumors to anti-PD-L1 therapy that is otherwise ineffective (9). Ex vivo analyses of 13 MSS CRC patient tumors have confirmed that dual blockade of PD-L1 and TIGIT (atezolizumab + tiragolumab) restores IFN-γ production and cytotoxic activity of tumor-infiltrating lymphocytes in 46% of MSS samples - an effect that single-agent atezolizumab failed to achieve - with TIGIT and PD-L1 co-expression on terminally exhausted T cells in MSS CRC validated by scRNA-seq and TCGA analyses (72). These findings motivate a triple combination strategy of COX2/PGE2 inhibition, PD-L1 blockade, and TIGIT antagonism that targets the root cause of TIGIT upregulation while simultaneously blocking its immunosuppressive downstream signaling. Importantly, combined blockade of PD-1 and LAG-3 (nivolumab + relatlimab) failed to produce meaningful responses in 59 evaluable pMMR/MSS CRC patients, with only 3 partial responses among 59 participants and no significant improvement in disease control rates (73). This negative result is instructive: it demonstrates that simply adding a second checkpoint inhibitor is insufficient to overcome the multi-cellular immunosuppressive network in MSS CRC, reinforcing the necessity of targeting upstream driving mechanisms rather than downstream inhibitory receptors. Notably, the gut microbiome has emerged as an unexpected modulator of MSS CRC immunotherapy responsiveness: Fusobacterium nucleatum, a CRC-associated bacterium, has been shown to facilitate anti-PD-1 therapy in MSS CRC through a butyric acid–HDAC3/8–TBX21 axis that transcriptionally represses PD-1 expression on CD8+ TILs, reactivating their effector functions and sensitizing MSS tumors to checkpoint blockade (74).

#### 8.1.2 TREM2+ TAM reprogramming

TREM2+ TAMs represent a high-priority therapeutic target in MSS CRC given their spatial enrichment in T cell-excluded zones and their role in suppressing productive immune synapses between MHC+ macrophages and TCF1+ T cells. CSF1R inhibition, which depletes TAMs or reprograms them toward an immunostimulatory phenotype, has been explored as a strategy to overcome myeloid-mediated immune suppression in CRC; single-cell transcriptomic analyses of CRC patients and murine models have identified distinct TAM subpopulations (SPP1+ and C1QC+ TAMs) with differential sensitivity to CSF1R blockade, with anti-CSF1R preferentially depleting SPP1+ immunosuppressive TAMs while sparing C1QC+ TAMs that support anti-tumor immunity (75). Four-agent blockade of TREM2, LAG-3, CTLA-4, and PD-1 achieves tumor clearance in over 70% of MMRp mouse models by restoring MHC+CXCL9+ macrophage–T cell interactions and generating immunological memory (33). A promising next-generation strategy, as discussed in a recent commentary on TREM2/IL-2 bispecific approaches, involves fusing TREM2-blocking antibodies to TME-restricted IL-2 variants (exemplified by MiTE-144) to simultaneously reprogram TAM immunosuppressive phenotypes and activate NK/CD8+ T cells while minimizing systemic IL-2 toxicity (76). Beyond TREM2, MS4A4A has been identified as a surface marker on immunosuppressive TAMs in CRC; anti-MS4A4A antibody treatment reprograms TAMs toward an immunostimulatory phenotype and restores CD8+ T cell-mediated anti-tumor immunity in preclinical CRC models, nominating MS4A4A as an additional myeloid checkpoint target (77).

SPP1 neutralization and IDO1 inhibition represent complementary TAM-targeting strategies that restore Tc1 differentiation and overcome tryptophan-mediated T cell dysfunction, respectively, and both show synergistic activity with PD-1 blockade in preclinical MSS CRC models (28, 32).

### 8.2 Targeting the CAF axis

#### 8.2.1 TGF-β pathway inhibition

Given the established role of stromal TGF-β signaling as a major upstream driver of CAF-mediated T cell exclusion in CRC, TGF-β receptor inhibitors (such as galunisertib, a TGFBR1 kinase inhibitor) in combination with PD-L1 blockade represent a clinically advanced strategy. The two Nature studies that established TGF-β as a major T cell exclusion mechanism provide the mechanistic basis for this combination (39, 64), and have provided the rationale for clinical exploration of TGF-β blockade plus PD-(L)1 inhibition in solid tumors. The bifunctional fusion protein M7824 (bintrafusp alfa), which simultaneously blocks PD-L1 and sequesters TGF-β, represents a single-agent approach to this combination.

#### 8.2.2 THBS2 targeting and senolytic strategies

THBS2 inhibition can dissolve the chemokine gradient blockade that enforces T cell exclusion in fibrotic MSS CRC, and the resulting T cell infiltration becomes therapeutically productive only when combined with checkpoint blockade - a finding that argues for a mandatory combination approach (40). Senolytic agents targeting senescent CAFs (navitoclax, dasatinib plus quercetin) block CD36-mediated lipid transfer to CD8+ T cells, restoring their cytotoxic function, while IL-1 receptor antagonism with anakinra disrupts the IL1R1+ iCAF–M2-TAM positive feedback loop and has entered clinical evaluation (38, 41). Targeting the MFAP2–LXRβ cascade with LXRβ antagonists or ERK1/2 inhibitors represents a more mechanistically novel approach that addresses the cross-cellular metabolic suppression relay (42).

### 8.3 Targeting Tregs with subpopulation precision

The Treg functional dichotomy in MSS CRC fundamentally reframes the therapeutic approach to Treg targeting: indiscriminate Treg depletion - as achieved by high-dose ipilimumab or CD25-targeted approaches - risks eliminating the anti-tumorigenic IL-10+ fraction and paradoxically accelerating tumor growth (51). CCR8-targeted depletion offers a precision alternative by selectively eliminating the pro-tumorigenic IL-10−/CCR8+ fraction while sparing IL-10+ Tregs. Preclinical studies have confirmed that CCR8-targeted immunotherapy induces dramatic changes to the intra-tumoral CD8+ T cell profile, with Treg depletion creating space for CD8+ T cell expansion and functional restoration (78). Multiple anti-CCR8 antibodies (including BMS-986340 and IPG7236) are in clinical development, and the CCR8/CTLA-4 bispecific antibody 2MW4691 achieves the dual benefit of selective Treg depletion and CD8+ T cell co-stimulatory disinhibition within a single molecule (54). These precision Treg-targeting strategies are predicted to be most effective in combination with anti-PD-1, as Treg depletion eliminates the suppressive brake while checkpoint blockade simultaneously reinvigorates T cell effector function.

### 8.4 Targeting MDSCs: trafficking blockade and differentiation induction

Inhibiting MDSC recruitment to MSS CRC through CXCR2 antagonism (SX-682) is being evaluated in combination with nivolumab in the STOPTRAFFIC-1 trial (NCT04599140), exploiting the IRF2–CXCL3–CXCR2 axis that drives KRAS-mutant MDSC trafficking. Differentiation-inducing agents - most notably all-trans retinoic acid (ATRA), which promotes MDSC maturation into immunostimulatory dendritic cells and macrophages - represent a complementary pharmacological approach to MDSC-mediated immunosuppression, and preclinical and mechanistic data suggest that ATRA combined with anti-PD-1 can convert the TME toward a T cell-permissive state, motivating clinical exploration in MSS CRC (57). PI3Kγ inhibition reprograms TAMs from an M2-like to M1-like phenotype and synergizes with checkpoint blockade in preclinical tumor models, supporting a myeloid-reprogramming strategy that may be relevant to MSS CRC where M2-like TAMs and MDSCs contribute to immune resistance (79). Analogous neoadjuvant immunotherapy strategies are now being prospectively evaluated in MSS CRC through the PURPLE trial (80); the conceptual rationale derives from dMMR proof-of-concept data (NICHE-3: 68% pCR with neoadjuvant nivolumab plus relatlimab in dMMR colon cancer, n=59 (81), which do not directly extend to MSS biology and therefore await dedicated MSS evidence.

### 8.5 Multi-target combination strategies and emerging directions

The multi-layered architecture of immunosuppression in MSS CRC makes clear that no single-target intervention will be sufficient to restore productive anti-tumor immunity. The strategies discussed below span a graded evidence base - from completed clinical trials (KEYNOTE-177 in MSI-H, botensilimab + balstilimab in MSS) and ongoing early-phase trials (PURPLE, STOPTRAFFIC-1, TORCH) to preclinical mechanistic studies (TREM2 quadruple blockade, CCR8 bispecifics) and rational combinations that remain conceptual - and should be interpreted accordingly. The most promising combination strategies target multiple layers simultaneously: TAM reprogramming (TREM2/CSF1R) combined with CAF targeting (THBS2/TGF-β/IL1R1) and ICI (anti-PD-1/PD-L1) addresses both the physical exclusion and functional impairment layers; COX2 inhibition combined with TIGIT and PD-L1 co-blockade targets the MSS-specific terminal exhaustion mechanism at its source; CCR8-targeted Treg depletion combined with anti-PD-1 addresses the active suppression layer while reinvigorating T cell effector function. The PURPLE trial (NCT05765994) is prospectively evaluating neoadjuvant atezolizumab plus tiragolumab in patients with resectable CRC liver metastases, providing both clinical efficacy data and matched pre- and post-treatment surgical specimens for mechanistic TME interrogation (80). The TORCH trial - a randomized phase II study of immunotherapy-based total neoadjuvant therapy for pMMR/MSS locally advanced rectal cancer - has provided important clinical data on the feasibility and activity of integrating checkpoint blockade into the neoadjuvant treatment of MSS CRC, with results informing the design of future combination strategies (82). A phase I trial of botensilimab (an Fc-enhanced anti-CTLA-4 antibody) plus balstilimab (anti-PD-1) in relapsed/refractory MSS metastatic CRC achieved a 17% objective response rate and 61% disease control rate (n=101), establishing proof-of-concept that enhanced CTLA-4 blockade combined with PD-1 inhibition can overcome MSS CRC resistance in select patients (83). An emerging cell-based strategy involves the adoptive transfer of CD160+CD8+ T cells - a subpopulation that maintains functional competence through the CD160–PI3K(p85α)–AKT–NF-κB–FcϵR1γ/4-1BB axis, resisting terminal exhaustion in PD-1 blockade-resistant CRC models (84); however, its efficacy in poorly immunogenic MSS-like models remains uncertain. Spatially guided therapeutic approaches, leveraging CODEX and Visium HD multiplex imaging to identify the specific layer of the three-tier immune-exclusionary niche that dominates in individual patients, represent the next frontier in precision immunotherapy for MSS CRC (**Table 2**).

**Table 2:** Therapeutic strategies for MSS CRC immune resistance, by evidence tier and target axis.

Strategy/agent	Target/mechanism	Cellular axis	Evidence tier	Key trial
Tier 1 — Published clinical or patient-sample efficacy data in CRC				
Botensilimab + balstilimab	Fc-enhanced anti-CTLA-4 + anti-PD-1	Treg + T cell	Phase I (R/R MSS metastatic CRC); ORR 17%, DCR 61% (n=101)	(83)
Nivolumab + relatlimab (in MSS CRC)	PD-1 + LAG-3 dual blockade	T cell	Phase II negative: 3 PR/59 evaluable pMMR; primary endpoint not met	(73)
Nivolumab + relatlimab (NICHE-3, dMMR)	PD-1 + LAG-3 neoadjuvant	T cell	Phase II in dMMR colon cancer; 68% pCR (n=59); conceptual precedent only — not MSS	(81)
Atezolizumab + tiragolumab (ex vivo)	PD-L1 + TIGIT	T cell + TAM downstream	Patient-tumor ex vivo mechanistic evidence: IFN-γ/cytotoxicity restored in 46% of 13 MSS CRC tumors (not a clinical trial)	(72)
Tier 2a — Ongoing early-phase trials in MSS/pMMR CRC or MSS-relevant CRC settings				
Atezolizumab + tiragolumab (PURPLE)	PD-L1 + TIGIT, neoadjuvant	T cell + TAM	Phase II neoadjuvant in resectable CRC liver metastases (MSI status not explicitly restricted; MSS-relevant rationale)	(80)
TORCH trial	Immunotherapy-based total neoadjuvant therapy	T cell + TME	Phase II randomized in pMMR/MSS locally advanced rectal cancer	(82)
Tier 2b — Cross-tumor clinical development with MSS CRC rationale (CRC-specific evidence limited)				
TGF-β pathway blockade + PD-(L)1	TGFBR1 kinase inhibitor (galunisertib as representative) + PD-(L)1	CAF + T cell	Clinical exploration in solid tumors; MSS CRC rationale from TGF-β exclusion studies (one urothelial, one CRC)	(39, 64)
Anakinra (IL-1Ra)	IL-1 receptor antagonist; disrupts iCAF–M2-TAM loop	CAF + TAM	Entered clinical evaluation (not necessarily MSS CRC-specific); broader TME-targeting development	(38)
Tier 3 — Preclinical mechanistic studies (often combined with ICI)				
COX2 inhibitors/EP2-EP4 antagonists + ICI	Suppress PGE2 → reduce TIGIT → reverse terminal exhaustion	TAM	Preclinical; mechanistic rationale strongest for MSS-associated PD-1+TIGIT+ terminal exhaustion	(9, 31)
Anti-TREM2 (quadruple blockade)	TREM2 + LAG-3 + CTLA-4 + PD-1	TAM	Preclinical: >70% tumor clearance in MMRp/MSS-like mouse models	(33)
MiTE-144 (TREM2/IL-2 bispecific)	Block TREM2 + TME-restricted IL-2 variant	TAM + NK/CD8	Preclinical commentary/next-generation design	(76)
Anti-MS4A4A	Myeloid surface marker depletion/reprogramming	TAM	Preclinical CRC models	(77)
Senolytics (navitoclax; dasatinib + quercetin)	Eliminate senescent CAFs; block CD36-lipid transfer	CAF	Preclinical CRC; clinical use in other indications	(41)
LXRβ antagonists/ERK1/2 inhibitors	Block MFAP2 → ITGB8 → ERK → CYP27A1 → LXRβ oxysterol axis	CAF → cancer → T cell	Preclinical	(42)
NNMT inhibitors (NNMTi) + ICI	Suppress CAF complement secretion → reduce MDSC recruitment	CAF + MDSC	Preclinical; validated in ovarian, breast, and colon (MC38)	(44)
2MW4691 (CCR8/CTLA-4 bispecific)	Treg depletion + CD8+ co-stimulatory disinhibition	Treg + T cell	Preclinical; related anti-CCR8 agents (BMS-986340, IPG7236) in early clinical development	(54)
ATRA + anti-PD-1	Induce MDSC maturation into DCs/macrophages	MDSC	Preclinical/mechanistic; clinical exploration proposed (not yet active)	(57)
Smad3 restoration/Mettl3 inhibition	Rescue MO-MDSC maturation blocked by m6A-mediated Smad3 destabilization	MDSC	Preclinical	(63)
YTHDF1 inhibition	Modulate m6A reader to enhance anti-PD-1 efficacy	MDSC/myeloid	Preclinical CRC	(61)
Tier 4 — Conceptual/hypothetical (limited or no direct MSS CRC validation)				
CD160+CD8+ T cell adoptive transfer	CD160–PI3K(p85α)–AKT–NF-κB–FcϵR1γ/4-1BB; resist exhaustion	T cell intrinsic	Preclinical in PD-1-resistant models; MSS efficacy uncertain	(84)
Microbiome-associated sensitization (F. nucleatum/butyrate axis)	HDAC3/8 inhibition → TBX21 induction → PD-1 transcriptional repression on CD8+ TILs	Microbiome → T cell intrinsic	Preclinical mechanism in MSS CRC; not yet a clinical strategy	(74)
Therapeutic TLS induction	Promote tertiary lymphoid structure formation to support Tpex niches	Stromal (Tpex maintenance)	Hypothetical; CCL19+ CAFs identified as biological clue, not yet a defined intervention	(24, 46)
Spatial-omics-guided patient stratification	CODEX/Visium HD/Xenium identification of dominant niche (TAM-/CAF-/Treg-dominated)	Cross-axis (biomarker)	Conceptual; biomarker development phase	(66)

Strategies are grouped by evidence maturity: published clinical or patient-sample efficacy data in CRC (Tier 1); ongoing early-phase trials specifically in MSS/pMMR CRC (Tier 2a); cross-tumor clinical development with MSS CRC mechanistic rationale but limited CRC-specific evidence (Tier 2b); preclinical mechanistic studies (Tier 3); and conceptual or hypothetical approaches (Tier 4). “Key trial/PMID” references the primary clinical or mechanistic study cited in the parent review. ICI, immune checkpoint inhibitor; pCR, pathologic complete response; ORR, objective response rate; DCR, disease control rate.

## 9 Conclusions and future perspectives

The evidence reviewed here establishes that CD8+ T cell exclusion, dysfunction, and exhaustion in MSS CRC are not cell-autonomous failures of T cell function but the product of a multi-cellular immunosuppressive network in which TAMs, CAFs, Tregs, and MDSCs cooperate through mechanistically distinct yet spatially and functionally integrated axes. Four key conclusions emerge from this synthesis. First, the MSS-specific PD-1+TIGIT+ terminal exhaustion phenotype is actively maintained by TAM-derived PGE2 - a mechanism absent in MSI-H disease - explaining why PD-1 monotherapy fails and why TIGIT co-blockade combined with COX2/PGE2 inhibition is a mechanistically rational strategy for MSS CRC specifically. Second, the Treg compartment in MSS CRC is functionally heterogeneous, with IL-10+ Tregs exerting unexpected anti-tumor effects through IL-17 suppression; this dichotomy demands precision targeting of the pro-tumorigenic CCR8+/IL-10− fraction rather than indiscriminate Treg depletion. Third, the immunosuppressive network is spatially organized, particularly in CRC liver metastases, into a stereotyped three-layer niche - mCAF outer belt, Tex/TSTR band, and SPP1+ TAM aggregates along the inner tumor-facing boundary - that physically enforces T cell exclusion and functional arrest, and whose architecture must be disrupted at multiple levels for therapeutic benefit. Fourth, the negative results of PD-1 plus LAG-3 co-blockade in MSS CRC confirm that adding checkpoint inhibitors to checkpoint inhibitors is insufficient; what is required is targeting the upstream cellular drivers that generate immune exclusion, effector dysfunction, and terminal exhaustion.

Several critical questions remain unresolved and define the agenda for future research. Whether a dominant cellular driver of immune exclusion, dysfunction, or exhaustion exists in individual MSS CRC patients - TAM-dominated versus CAF-dominated versus Treg-dominated - and whether such patient subgroups can be identified prospectively using spatial multi-omics biomarkers (FAP/SPP1/CCR8/THBS2 expression profiles) is a question of immediate clinical relevance. The dynamic evolution of the immunosuppressive network in response to treatment - including the compensatory upregulation of alternative suppressive axes when one is pharmacologically targeted - will need to be characterized through longitudinal sampling of matched pre- and post-treatment specimens, as the PURPLE trial is designed to enable (80). The anti-tumor function of IL-10+ Tregs raises the intriguing possibility that this population could be therapeutically harnessed rather than merely preserved - for example, by redirecting their IL-10 production to suppress pro-tumorigenic IL-17 signaling in a spatially controlled manner. The Smad3–m6A axis in MO-MDSCs (63) and the ID3-dependent developmental origin of Tpex cells (21) represent newly identified molecular nodes that may be amenable to pharmacological intervention to restore myeloid maturation and sustain the progenitor T cell pool, respectively. The prognostic and predictive value of TLS density in MSS CRC - and the possibility of therapeutically inducing TLS formation to sustain the Tpex compartment - represents a further avenue of investigation (24).

Looking forward, the convergence of spatial multi-omics technologies - CODEX, Visium HD, Xenium, and co-detection by indexing - with patient-derived organoid co-culture systems that recapitulate multi-cellular TME interactions will provide the experimental infrastructure needed to systematically dissect the relative contributions of each cellular axis in individual patients and to screen combination therapeutic strategies in a patient-specific manner. Artificial intelligence-assisted spatial immunomics analysis, capable of identifying the three-layer niche architecture and quantifying its constituent cell populations from routine clinical specimens, offers a path toward biomarker-driven patient stratification that could guide the selection of combination immunotherapy regimens. Ultimately, the framework presented in this Review - multicellular orchestration of CD8+ T cell exclusion, dysfunction, and exhaustion through four integrated axes operating across four functional layers within spatially organized niches - provides both a mechanistic explanation for the failure of current immunotherapy in MSS CRC and a rational blueprint for the multi-target combination strategies that will be required to overcome it.
